# Self-rated health among teachers: prevalence, predictors, and impact on absenteeism, presenteeism, and sick leave

**DOI:** 10.47626/1679-4435-2021-619

**Published:** 2021-12-30

**Authors:** Diogo Henrique Constantino Coledam, Gustavo Aires de Arruda, Edineia Aparecida Gomes Ribeiro, Francys Paula Cantieri

**Affiliations:** 1 Educação Física, Instituto Federal de Educação, Ciência e Tecnologia de São Paulo, Boituva, SP, Brazil.; 2 Enfermagem, Universidade de Pernambuco, Recife, PE, Brazil.; 3 Educação Física, Universidade Federal do Mato Grosso do Sul, Corumbá, MS, Brazil.; 4 Educação Física, Universidade de Pernambuco, Recife, PE, Brazil.

**Keywords:** self-assessment, health status, occupational exposure, lifestyle, health, autoavaliação, estado de saúde, exposição ocupacional, estilo de vida, saúde

## Abstract

**Introduction:**

Self-rated health is an important indicator of health in the population, but among teachers, predictive sociodemographic, occupational, and health variables and the impact of self-rated health on absenteeism, presenteeism, and sick-leave are unknown.

**Objectives:**

The objectives of this study were to determine the prevalence of and factors associated with poor self-rated health among elementary school teachers and to investigate whether poor self-rated health can predict absenteeism, presenteeism, and sick leave.

**Methods:**

The sample comprised 493 elementary school teachers from Londrina, Paraná, Brazil. A self-report questionnaire was used to assess self-rated health and sociodemographic, occupational, and lifestyle factors, health indicators, chronic diseases, disabilities, and number of days of absenteeism, presenteeism, and sick leave. Poisson regression was used to estimate prevalence ratios and rate ratios.

**Results:**

The prevalence of poor self-rated health was 16.4% and the variables positively associated with this outcome were health insurance (prevalence ratio = 2.35), inadequate school infrastructure (prevalence ratio = 1.56), physical activity (prevalence ratio = 0.60), poor perceived fitness (prevalence ratio = 2.44), voice disorders (prevalence ratio =1.46), common mental disorders (prevalence ratio = 1.74), emotional exhaustion (prevalence ratio = 1.61), low personal accomplishment (prevalence ratio = 1.64), chronic disease (prevalence ratio = 2.39), and disability (prevalence ratio = 1.57). Poor self-rated health was positively associated with both absenteeism (rate ratio=1.71) and presenteeism (rate ratio = 1.74).

**Conclusions:**

Occupational and individual characteristics associated with impaired physical and mental health should be targeted to improve self-rated health among teachers. Furthermore, a single question on self-rated health is a useful tool for monitoring and preventing absenteeism and presenteeism among teachers.

## Resumo

**Introdução:**

A autopercepção de saúde é um importante indicador de saúde na população; no entanto, em professores, as variáveis preditoras sociodemográficas, ocupacionais e de saúde, bem como o impacto da autopercepção de saúde no absenteísmo, presenteísmo e licenças médicas são desconhecidos.

**Objetivos:**

Determinar a prevalência e os fatores associados à autopercepção de saúde ruim em professores do ensino fundamental e investigar se é preditora de absenteísmo, presenteísmo e licenças médicas.

**Métodos:**

A amostra foi composta por 493 professores da cidade de Londrina, Paraná, Brasil. Um questionário de autorrelato foi utilizado para avaliar a autopercepção de saúde; os fatores sociodemográficos, ocupacionais e de estilo de vida; os indicadores de saúde; a presença de doenças crônicas e incapacidade; e as taxas de absenteísmo, presenteísmo e licenças médicas. Foi utilizada regressão de Poisson para estimar as razões de prevalência e as razões de taxa.

**Resultados:**

A prevalência da autopercepção de saúde ruim foi de 16,4%, tendo as seguintes variáveis apresentado associação positiva com o desfecho: plano de saúde (razão de prevalência = 2,35), infraestrutura escolar inadequada (razão de prevalência = 1,56), atividade física (razão de prevalência = 0,60), percepção de baixa aptidão física (razão de prevalência = 2,44), distúrbios vocais (razão de prevalência = 1,46), transtornos mentais comuns (razão de prevalência = 1,74), exaustão emocional (razão de prevalência = 1,61), baixa realização profissional (razão de prevalência = 1,64), doença crônica (razão de prevalência = 2,39) e incapacidade (razão de prevalência = 1,57). A autopercepção de saúde ruim apresentou associação positiva com o absenteísmo (razão de taxas = 1,71) e presenteísmo (razão de taxas =1,74).

**Conclusões:**

Características ocupacionais e individuais associadas à deterioração da saúde física e mental devem ser foco de ações de prevenção, a fim de melhorar a autopercepção de saúde docente. Uma única pergunta sobre a autopercepção de saúde é uma ferramenta útil para monitorar e prevenir o absenteísmo e presenteísmo docente.

## INTRODUCTION

Self-rated health is an internationally validated and reliable global measure of health status and can be assessed using a single question.^1^ In population studies, it is considered the most feasible, inclusive, and informative measure of health status.^1^ There is a considerable body of data showing that predictors of poor self-rated health among adults include age, socioeconomic status, a variety of lifestyle characteristics, physical and mental disorders, the presence of a variety of chronic diseases, health service use, medication use, and mortality.^1-6^

Work-related variables should be considered when analyzing self-rated health among adults. Although work can contribute to life satisfaction, a poor work environment can result in the opposite effect.^7^ In view of this, the teaching profession is highlighted in the literature, focusing on the association between working conditions and health risks, due to the high prevalence of physical and mental disorders,^8,9^ as well as frequent dissatisfaction with the work environment.^10^ As a consequence, both health status and occupational characteristics negatively affect work ability^11^ and personal life.^12^ Another result of the severity of multiple health disorders and a poor work environment is the frequent occurrence of absenteeism and presenteeism among teachers.^13,14^ Both variables have serious implications for personal life, health, and productivity, and negatively affect student achievement.^15,16^

With regard to self-rated health among Brazilian teachers, 14.3 to 27% of teachers perceived their health as poor/regular.^17-19^ Recent evidence from the EDUCATEL study indicates that self-rated health is inversely associated with pressure to work.^18^ Although descriptive data is available in the literature, limited information is available about sociodemographic, occupational, lifestyle, and health-related predictors of self-rated health among teachers and the specific characteristics of this profession prevent generalization of results from the general population.^2-6^ In addition, it is unknown if poor self-rated health is associated with absenteeism, presenteeism, and sick leave, and, thus, how self-rated health can be used to prevent and monitor these relevant teachers’ health outcomes.

Given the above, the objectives of this study were: 1) to determine the prevalence of and factors associated with poor self-rated health among elementary school teachers; 2) to investigate whether poor self-rated health can predict days of absenteeism, presenteeism, and sick leave.

## METHODS

The present study is part of a project aimed at investigating sociodemographic, work condition, and health risk variables associated with health service use, medication consumption, absenteeism, and presenteeism among elementary school teachers. Further information can be found elsewhere.^8,13,20^ This is a cross-sectional study conducted with a probabilistic sample of teachers from Londrina, Paraná, Brazil, in 2014. The study was approved by the Ethics Committee for Research involving human beings at the Universidade Estadual de Londrina (Protocol 118/2014). This study complies with the guidelines set out in Brazilian National Health Council Resolution nº 466/2012.

Sample size was calculated using OpenEpi 3.0 software with the following parameters: N = 2,500, 50% outcome prevalence, 5% sample error, 95% confidence interval (95%CI), design effect of 1.5, and 20% sample loss. For the present study, a sample size of 430 teachers was required, but a larger sample (n = 595) was recruited because of other aims of the project. The sample was stratified according to each region of the city (north 32.8%, south 20%, east 20.3%, west 22.1%, and center 4.8%). The inclusion criteria adopted were: a) having been a teacher in municipal schools for at least 1 year and be working in an elementary school; b) not being retired or on medical leave during data collection; c) not having been work relocated (i.e., teacher working as a secretary or in administration). Teachers with incomplete data were excluded from the sample.

After authorization was granted by the Municipal Education Department, all of the city’s schools were visited (n = 74) to present the project to their principals. At schools where the principals agreed to participate (n = 63), another visit was performed to present the project aims and procedures and to invite teachers to participate in the study and distribute informed consent forms. A data collection day was scheduled and a second data collection was conducted for those who were absent on the first date. All procedures were performed at the school at which the teachers were enrolled and data collection was conducted by the project coordinator.

All variables were assessed using a printed self-report questionnaire. The dependent variable was poor self-rated health for study objective 1, and absenteeism, presenteeism, and sick leave days for study objective 2. Self-rated health was evaluated using the question “How do you rate your health status?”, with the following response options: “great”, “very good”, “good”, “regular”, and “poor”. Open questions were used to assess the following outcomes: absenteeism, “In the last year, how many days were you absent from work through your own decision because of your health”; presenteeism, “In the last year, while working, how many days did you not perform your activities as usual due to some health problem”; and sick leave, “In the last year, how many days did you need to be on medical sick leave?”.^13^

The independent variables were allocated to five categories, as follows: sociodemographic (sex, age, income, health insurance, and children); occupational (length of employment, school infrastructure, violence at school, work shift, number of schools worked at, extra work, occupational stress, and low job support); lifestyle (physical activity, tobacco use, and alcohol consumption); health (poor perceived fitness, voice disorders, common mental disorders, problems related to dust, emotional exhaustion, depersonalization, personal accomplishment, recurrent musculoskeletal symptoms); and disease and disability (chronic disease and disability due to musculoskeletal disorders).

Sex, age, number of children, length of employment, work shifts, number of schools worked at, and unpaid extra work were assessed using open questions. Income was estimated using the Brazilian Association of Polling Companies questionnaire. Physical activity was estimated using the International Physical Activity Questionnaire, considering all dimensions. The cut-off used was 150 minutes of moderate to intense physical activity a week. The presence of common mental disorders was evaluated by the Self Report Questionnaire^21^ and the cut-off adopted was 7/8. The Maslach Burnout Inventory was used to assess burnout^22^ and the Job Stress Scale was used for occupational stress and low job support.^23^ The Nordic Musculoskeletal Questionnaire^24^ was used to assess recurrent musculoskeletal symptoms in the neck, shoulder, and upper or back regions (assessing symptoms both in the previous 12 months and in last 7 days), and disability.

Closed questions were asked to assess the following variables: health insurance, “Do you pay for health services?”, with answers grouped as “yes” (those who reported private or employer health insurance) or “no” (those who reported using only the public health system”; school infrastructure, “Do you consider the infrastructure of your school to be appropriate?” (a list of items that should be considered was presented: noise, temperature, lighting, cleaning, ventilation, size, and furniture) with response options “yes”, “no”, and “partially”; physical violence, “Have you ever suffered physical violence at work?” with the response options “yes” and “no”; tobacco use, “Have you smoked at least 100 cigarettes in your entire life?”, “yes” and “no”; alcohol consumption (binge drinking), “Usually, how many times in a normal month do you consume ≥ 5 doses of the drinks below (men) or ≥ 4 doses (women) in less than 2 hours? (doses of beer, wine, and distilled drinks were presented in milliliters); voice disorders, “Do you have a frequent voice-related problem? “yes”, “no”; problems related to chalk powder, “Do you have a frequent problem with dust or chalk powder?”, “yes”, “no” for each problem (nasal stuffiness, eye irritation, rhinitis, coryza, cough, and skin problems). Perceived physical fitness: “Do you have difficulty to perform work (domestic or formal) that involves physical effort?”, with the response options “no”, “low difficulty”, and “high difficulty” (those who reported “low” and “high difficulty” were classified as having poor fitness); chronic disease, “Has a doctor or psychologist reported that you have any of the following chronic diseases?” with answer options “yes” and “no” for a list of categories of chronic diseases (cardiometabolic, psychological, orthopedic, respiratory, gastrointestinal, nervous system, and cancer).^8,20^

Descriptive statistics were calculated using absolute and relative frequencies and rates of absenteeism, presenteeism, and sick leave days. Bivariate analysis of the associations between independent variables and poor self-rated health were conducted using the chi-square test. Variables associated at p < 0.20 according to the chi-square test were included in a multivariate model using Poisson regression with robust variance estimation to estimate the prevalence ratio (PR) and confidence interval of 95% (95%CI) with five hierarchical levels: level 1 (sociodemographic); level 2 (occupational); level 3 (lifestyle); level 4 (health disorders), and level 5 (chronic disease and disability). Independent variables were retained in the final models when their associations were significant (p < 0.05) or if they affected the magnitude of the other associations. Poisson regression was also employed to perform multivariate analysis to estimate the rate ratios (RR) for the associations between poor self-rated health and absenteeism, presenteeism, and sick leave. All analyses were conducted using Stata 11.0 software (StataCorp, College Station, TX, USA).

## RESULTS

A total of 595 teachers agreed to participate in the study and performed all procedures. However, 102 were excluded from the analysis due to incomplete information for at least one variable on the questionnaire.

The prevalence of poor self-rated health in the sample analyzed was 16.4%. Tables 1 to 3 present the profile of the sample according to the variables analyzed. Higher proportions of participants had the following characteristics: female, medium or high income, health insurance, children, length of employment ≥ 20 years, adequate school infrastructure, had not experienced violence, two or three work shifts, worked at one school, did not perform extra work, did not report high stress, and did not report low job support. Furthermore, higher proportions of teacher also achieved the recommended physical activity level and did not have poor perceived physical fitness, tobacco use, common mental disorder, voice disorder, emotional exhaustion, reduced personal accomplishment, depersonalization, recurrent musculoskeletal symptoms, or chronic diseases and disabilities, while most teachers reported problems related to dust. Distributions were similar for age and presence of chronic disease.

**Table 1:** Association between sociodemographic variables and poor self-rated health

Variables	All* n (%)	Poor self-rated health (%)	Crude PR (95%CI)	Adjusted^†^ PR (95%CI)
Level 1				
Sex		p = 0.193		
Male	27 (5.5)	7.4	Reference	Reference
Female	466 (94.5)	17.0	2.28 (0.59-8.82)	2.03 (0.53-7.67)
Age (years)		p = 0.185		
< 40	234 (47.5)	14.1	Reference	Reference
≥ 40	259 (52.5)	18.5	1.31 (0.87-1.97)	1.23 (0.82-1.86)
Income		p = 0.296		
Low	79 (16.0)	12.7	Reference	Reference
Medium	214 (43.4)	16.4	1.29 (0.67-2.48)	-
High	200 (40.6)	18.0	1.42 (0.74-2.72)	-
Health insurance		p = 0.009		
No	94 (19.1)	7.4	Reference	Reference
Yes	399 (80.9)	18.5	**2.49 (1.18-5.23)**	**2.35 (1.11-4.99)**
Children		p = 0.302		
No	141 (28.6)	19.1	Reference	Reference
Yes	352 (71.4)	15.3	0.80 (0.52-1.21)	-

Bold denotes significance at p < 0.05.

95%CI = 95% confidence interval; PR = prevalence ratio.

* Distribution of sample according to the independent variables analyzed.

† Adjusted for variables at the same level.

**Table 3:** Association between lifestyle, health variables, disability, and poor self-rated health

Variables	All* n (%)	Poor self-rated health (%)	Crude PR (95%CI)	Adjusted^†^ PR (95%CI)
Level 3				
Recommended physical activity level		p = 0.013		
No	102 (20.7)	24.5	Reference	Reference
Yes	391 (79.3)	14.3	**0.58 (0.38-0.88)**	**0.60 (0.40-0.90)**
Tobacco use		p = 0.548		
No	430 (87.2)	16.0	Reference	Reference
Yes	63 (12.8)	19.0	1.18 (0.68-2.06)	-
Alcohol consumption		p = 0.079		
No	328 (66.5)	18.3	Reference	Reference
Yes	165 (33.5)	12.1	0.69 (0.43-1.10)	-
Level 4				
Poor perceived fitness		p < 0.001		
No	289 (58.6)	8.3	Reference	Reference
Yes	204 (41.4)	27.9	**3.36 (2.16-5.23)**	**2.44 (1.55-3.84)**
Voice disorders		p = 0.005		
No	317 (64.3)	12.9	Reference	Reference
Yes	176 (35.7)	22.7	**1.75 (1.18-2.60)**	**1.46 (1.02-2.18)**
Common mental disorders		p < 0.001		
No	317 (64.3)	10.4	Reference	Reference
Yes	176 (35.7)	30.4	**2.91 (1.96-4.32)**	**1.74 (1.11-2.72)**
Problems related to dust		p = 0.001		
No	143 (29.0)	10.5	Reference	Reference
One	159 (32.3)	12.6	1.19 (0.63-2.25)	-
≥ Two	191 (38.7)	24.1	**2.29 (1.33-3.94)**	-
Emotional exhaustion		p < 0.001		
No	370 (75.1)	11.9	Reference	Reference
Yes	123 (24.9)	30.1	**2.52 (1.71-3.72)**	**1.61 (1.06-2.46)**
Depersonalization		p = 0.754		
No	354 (71.8)	16.1	Reference	Reference
Yes	139 (28.2)	17.3	1.07 (0.69-1.65)	-
Reduced personal accomplishment		p < 0.001		
No	358 (72.6)	12.3	Reference	Reference
Yes	135 (27.4)	27.4	**2.22 (1.50-3.29)**	**1.64 (1.08-2.48)**
Recurrent musculoskeletal symptoms				
Neck		p < 0.001		
No	318 (64.5)	11.6	Reference	Reference
Yes	175 (35.5)	25.1	**2.16 (1.45-3.21)**	-
Level 5				
Chronic diseases		p < 0.001		
No	223 (45.2)	6.3	Reference	Reference
One	109 (22.1)	19.3	**3.06 (1.56-6.03)**	**2.22 (1.16-4.25)**
≥ Two	161 (32.7)	28.6	**4.55 (2.50-8.27)**	**2.39 (1.32-4.32)**
Disability		p < 0.001		
No	317 (64.3)	10.4	Reference	Reference
Yes	176 (35.7)	27.3	**2.61 (1.68-4.08)**	**1.57 (1.02-2.43)**

Bold denotes significance at p < 0.05.

95%CI = 95% confidence interval; PR = prevalence ratio.

* Distribution of sample according to independent variables analyzed.

† Adjusted for variables of the same level plus variables from previous level.

The bivariate analysis of the associations between independent variables and negative self-rated health is presented in Tables 1 to 3. In level 1, a higher prevalence of negative self-rated health was found in those with health insurance. In level 2, inadequate school infrastructure and performing extra work were positively associated with negative self-rated health. Physical activity exhibited a negative association (level 3) and positive associations were found with poor physical fitness perception, voice disorders and common mental disorders, problems related to dust, emotional exhaustion, reduced personal accomplishment, and recurrent musculoskeletal symptoms (level 4). Both variables in level 5 were positively associated with the outcome. In all cases p < 0.05.

The multivariate analysis revealed that health insurance, PR = 2.35 (Table 1), adequate school infrastructure, PR = 1.56 (Table 2), physical activity (PR = 0.60), poor perceived fitness PR = 2.44, voice disorders PR = 1.46, common mental disorders PR = 1.74, emotional exhaustion PR = 1.61, reduced personal accomplishment PR = 1.64, chronic disease PR = 2.22 to 2.39, and disability due to musculoskeletal disorder PR = 1.57 (Table 3) were the significant predictors of negative self-rated health.

**Table 2:** Associations between occupational variables and poor self-rated health

Variables	All* n (%)	Poor self-rated health (%)	Crude PR (95%CI)	Adjusted^†^ PR (95%CI)
Level 2				
Length of employment (years)		p = 0.132		
Up to 9	129 (26.2)	15.5	Reference	Reference
10 to 19	162 (32.9)	11.7	0.75 (0.42-1.35)	-
≥ 20	202 (41.0)	20.8	1.34 (0.82-2.17)	-
School infrastructure		p = 0.018		
Adequate	384 (77.9)	14.3	Reference	Reference
Not adequate	109 (22.1)	23.9	**1.66 (1.09-2.52)**	**1.56 (1.02-2.39)**
Violence at school		p = 0.578		
No	377 (76.5)	15.9	Reference	Reference
Yes	116 (23.5)	18.1	1.13 (0.72-1.78)	-
Work shifts		p = 0.798		
One	152 (30.8)	15.8	Reference	Reference
Two/three	341 (69.2)	16.7	1.05 (0.68-1.63)	-
Number of schools		p = 0.680		
One	272 (55.2)	15.8	Reference	Reference
≥ two	221 (44.8)	17.2	1.08 (0.72-1.62)	-
Extra work		p = 0.047		
No	355 (72.0)	14.4	Reference	Reference
Yes	138 (28.0)	21.7	1.51 (1.01-2.27)	-
Occupational stress		p = 0.262		
No	352 (71.4)	17.6	Reference	Reference
Yes	141 (28.6)	13.5	0.76 (0.47-1.23)	-
Low job support		P = 0.220		
No	280 (56.8)	14.6	Reference	Reference
Yes	213 (43.2)	18.8	1.28 (0.86-1.90)	-

Bold denotes significance at p < 0.05.

95%CI = 95% confidence interval; PR = prevalence ratio.

* Distribution of sample according to independent variables analyzed.

† Adjusted for variables of the same level plus variables from previous level; a high demand and low control of work.

Crude and adjusted analyses of the association between negative self-rated health and absenteeism, presenteeism, and sick leave are presented in Table 4. Teachers who reported poor self-rated health presented a higher rate ratio of absenteeism RR = 2.53 and 1.71, and presenteeism RR = 3.44 and 1.74, in both the bivariate and multivariate analyses respectively (p < 0.05). This did not occur for sick leave (p > 0.05).

**Table 4:** Association between self-rated health and absenteeism, presenteeism, and sick-leave

	Absenteeism days		Presenteeism days		Sick-leave days	
	Rate	Crude RR (95%CI)	Rate	Crude RR (95%CI)	Rate	Crude RR (95%CI)
Self-rated health						
Good	2.59	Reference	3.60	Reference	3.91	Reference
Poor	6.57	**2.53 (1.56-4.09)**	12.43	**3.44 (1.67-7.10)**	5.95	1.51 (0.78-2.92)
	**Adjusted RR (95%CI)**		**Adjusted RR (95%CI)**		**Adjusted RR (95%CI)**	
Self-rated health						
Good	Reference		Reference		Reference	
Poor	**1.71 (1.16-2.52)**		**1.74 (1.01-2.99)**		0.86 (0.44-1.71)	

Bold denotes significance at p < 0.05.

Models adjusted for: sex, age, income, school infrastructure, violence, low job support, high stress, common mental disorders, musculoskeletal symptoms, physical activity, voice disorders, problems related to dust, burnout, chronic disease and disability.

95%CI = 95% confidence interval; RR = rate ratio.

## DISCUSSION

To our knowledge, this is the first study to determine the predictors of poor self-rated health and its impact on absenteeism, presenteeism, and sick leave days among teachers. Health insurance, inadequate school infrastructure, poor physical fitness, voice disorders, common mental disorders, emotional exhaustion, low personal accomplishment, and chronic disease and disability were positively associated with poor self-rated health. Furthermore, poor self-rated health was associated with more days of absenteeism and presenteeism, but not with sick leave days.

The prevalence of poor self-rated health in the present sample was 16.4%, similar to rates found in two other studies 13.3%^17^ and 19.9%,^19^ but different from the 27% prevalence found in a more recent study.^18^ Two aspects could explain the differences. First, the studies with a similar prevalence are from southern regions of Brazil, while the EDUCATEL study has a nationwide sample. The heterogeneity of sociocultural and occupational factors in Brazil may contribute to the difference in prevalence. Another aspect is that in the present study poor self-perceived health was made up of the group of teachers who answered that their health was “poor” or “regular”, as commonly occurs in the literature.^2-4^ Since two of the earlier studies used the same answer structure,^17,19^ it was possible to compare the results for the same construct. However, in the EDUCATEL study,^18^ self-rated health was assessed with different answer options and three categories were clustered to categorize poor and regular answers (very poor, poor, regular). It is probable that these reasons contributed to the finding of a higher prevalence of poor self-rated health.

Contrary to the extensive descriptions in the literature,^2-4^ age was not associated with poor self-rated health among teachers. Older teachers had a slightly higher prevalence of poor self-rated health, but this was insufficient to achieve statistical significance. This indicates that among teachers, poor self-rated health increases little with age, in contrast with what occurs in the general population, where older people commonly present more than double the prevalence of poor self-rated health compared to young people.^2-4^ The only sociodemographic variable positively associated with poor self-rated health was health insurance. In general, a better health profile is observed in those who have health insurance, but the associations are not homogeneous across a variety of health risks or diseases over the years.^25^ For example, the prevalence rates of chronic diseases according to whether or not the subject has health insurance are quite similar even when statistical significance is attained.^25^ However, higher differences (two-fold in some years) have been described for poor self-rated health in individuals who do not have health insurance,^25^ which is opposite to the present result. Comparison of the present study with those conducted in the general population is difficult because of specific characteristics related to samples of teachers. To illustrate this point, 80.9% of teachers reported having private health insurance (private or from an employer), a prevalence that is considerably higher than in the general population (25.3%).^26^ It is not possible to affirm the direction of causality of this association with the present data, but a plausible hypothesis is that teachers with poor self-rated health probably have health insurance to treat their health disorders. One finding that supports this hypothesis is that teachers with health insurance report higher use of medications for orthopedic disorders.^20^

In the occupational level, only inadequate school infrastructure was associated with poor self-rated health. School infrastructure is a variable that encompasses a wide range of factors such as noise, temperature, lighting, cleaning, ventilation, size, and furniture, as described previously.^8,13,27^ Despite the absence of information regarding poor self-rated health among teachers, school infrastructure has been associated with poor musculoskeletal health,^8^ simultaneous medication use,^20^ and absenteeism, presenteeism, and sick leave days.^13^ Similarly, it has been reported that there is a positive association between perceived school physical environment and overall quality of life, and physical and psychological domains among Brazilian teachers^10^ and the same was observed in Chinese teachers for the physical domain of quality of life.^27^ A recent study demonstrated that self-rated health is inversely associated with pressure to work,^18^ however the analysis was not adjusted for potential confounders. The present results are curious, since a variety of other characteristics that are associated with health impairment in teachers were analyzed in the occupational level, such as experience of violence, overload, high stress, and low job support. These data indicate that self-rated health among teachers is affected to a lesser extent by occupational factors and mostly by their objective health condition. However, it is important to state that the occupational environment is a relevant aspect in the etiology of a range of work-related diseases in this population.

It was expected that some health disorders would present an association with poor self-rated health, but it was unknown which would be the best predictors among teachers. The association between poor self-rated health and physical activity,^2,3,28^ poor perceived fitness,^28^ mental health,^29^ voice disorders, and chronic disease and disability^2,4^ are in line with previous studies conducted with samples from the general adult population. These findings indicate that, among teachers, the variables that cause physical or mental suffering or have perceptible symptoms impact on self-rated health. Additionally, knowledge about objective health indicators could be an aspect that explains the association, since some health disorders are diagnosed by a physician (i.e., chronic diseases). Furthermore, since teachers have high levels of schooling and discussion about teachers’ health is common among peers, institutions, and academies, it is probable they have good understanding about health components^1^ and are able to express it through the self-rated health scale used. The same could be true with regard to physical activity, since the benefits are widely disseminated in the media and by physical education professionals.

The health indicators alcohol consumption, tobacco use, and musculoskeletal symptoms were not associated with poor self-rated health. Although the mechanisms of this absence of association were not investigated in this study, it is possible to hypothesize the reasons. Alcohol consumption was assessed by binge drinking in the previous month, and since this is highly prevalent among the Brazilian population,^30^ the perception of harmful effects may be underestimated. It is important to state that results regarding alcohol consumption are in line with previous literature.^4,5^ With regard to tobacco use, participants were classified as users if they reported smoking at least 100 cigarettes in their entire life, since the low prevalence of current smokers made the analysis impractical. As a consequence, teachers with low cigarette consumption during their life can disregard the harmful effect of tobacco on their health. Another variable that was not associated with poor self-rated health was musculoskeletal symptoms. This result is curious since this is a relevant and highly prevalent health indicator, and the neck and shoulders were the most affected regions of the body due to the type of work performed by teachers. Although it was not associated with poor self-rated health, this variable should be prevented or managed as severity of musculoskeletal pain is associated with chronic disease, disability, and common mental disorders among teachers.^8^

The second aim of the present study was to investigate the association between poor self-rated health and absenteeism, presenteeism, and sick leave. Teachers have high prevalence and high rates of absenteeism and presenteeism.^13,14^ These outcomes are interrelated and have complex etiology involving environmental, person-related, work-related, and organizational variables.^15^ The literature reports that a poor occupational environment, lifestyle, physical and mental health, chronic disease, and health services used are associated with higher rates of both absenteeism and presenteeism among Brazilian teachers.^13,14^ One hypothesis raised is that the association of poor self-rated health with absenteeism and presenteeism occurs throughout the health variables described. However, the present results show that poor self-rated health is associated with both absenteeism and presenteeism independently, since presence of health disorders was controlled in the multivariate analysis. These results suggest that teachers are aware of the characteristics that affect their current health status and, consequently, their absenteeism and presenteeism. This is relevant because the accuracy of individual information about body condition results in better prognostic value of self-rated health.^1^ Although this affirmation was stated in the context of mortality, it can probably be applied in the present study. These results have relevant practical implications. Monitoring the self-rated health of teachers using a simple instrument can be used to prevent or predict absenteeism and presenteeism among teachers, and, consequently, losses related to the personal, health, and occupational dimensions of these professionals.

Some limitations of the present study should be discussed. Although the selection of study variables and analyses considered the complex etiology of poor self-rated health, the causality of the relationships could not be assessed in the present study. All variables were assessed using a self-report questionnaire which may be influenced by recall bias. Furthermore, self-assessment depends on understanding what health is, the components of health, how different aspects can impair health, and how to evaluate health as a global construct.^1^ Two aspects that minimizes this limitation in the current study is that the sample was composed of participants with high school educational level and the questionnaire was constructed from previously validated instruments. Despite its limitations, the probabilistic sampling, and multiple analysis considering relevant variables related to occupational factors and health of teachers are strengths of this study. Since there are similarities in the public Brazilian educational system, with respect to legislation and teaching working conditions, the results of this study have potential for generalization to other regions.

## CONCLUSIONS

The prevalence of poor self-rated health was 16.4%. Health insurance, inadequate school infrastructure, physical activity, poor self-perceived fitness, voice disorders, common mental disorders, emotional exhaustion, reduced personal accomplishment, chronic disease, and disability were predictors of poor self-rated health among the sample studied. Poor self-rated health was associated with greater absenteeism and presenteeism, but not with sick leave days. Occupational and individual characteristics associated with impaired physical and mental health should be targeted to improve self-rated health among teachers. Furthermore, a single question on self-rated health is a useful tool to monitor and prevent absenteeism and presenteeism among teachers; variables that are a consequence of the severity of health disorders and considerably affect teachers’ personal and professional lives.
